# EP3 is the only prostaglandin E receptor required for lipopolysaccharide-induced fever

**DOI:** 10.1016/j.bbih.2026.101344

**Published:** 2026-09-03

**Authors:** Unn Kugelberg, Anna Erkstam, Kiseko Shionoya, Anders Blomqvist

**Affiliations:** Division of Neurobiology, Department of Biomedical and Clinical Sciences, Faculty of Medicine and Health Sciences, Linköping University, Linköping, 581 85, Sweden

**Keywords:** Fever, Prostaglandin E2, EP3 receptor, Lipopolysaccharide, Hypothalamus, Telemetry

## Abstract

Fever induced by systemic inflammation is mediated by prostaglandin E2 (PGE2), but the identity of the prostaglandin E (EP) receptor subtype responsible for the febrile response remains debated. Although EP3 receptors have been strongly implicated in fever generation, studies using pharmacological approaches and receptor-deficient mice have also suggested involvement of EP1 and EP4 receptors. Because previous studies have differed substantially with regard to experimental conditions, including ambient temperature, route of immune challenge, and injection-associated stress responses, we systematically compared the febrile response to lipopolysaccharide (LPS) in mice lacking EP1, EP2, EP3, or EP4 receptors under standardized physiological conditions. Body temperature was recorded by telemetry in mice housed at thermoneutrality and injected intravenously with LPS (30 μg/kg) through indwelling jugular catheters, permitting remote injections with minimal disturbance to the animals. Mice with global deletion of EP1, EP2, or EP3, as well as mice with nervous system-directed deletion of EP4, all maintained on a C57BL/6 background, were examined together with wild-type littermates. Wild-type mice displayed a characteristic multiphasic fever following LPS administration. Mice lacking EP1 or EP2, or with nervous system-directed deletion of EP4, exhibited febrile responses indistinguishable from those of their wild-type controls. In contrast, mice lacking EP3 failed completely to develop fever and instead displayed a pronounced hypothermic response immediately following LPS injection. These findings identify EP3 as the only prostaglandin E receptor with a non-redundant role in LPS-induced fever and support the concept that EP3-dependent signaling constitutes the final common pathway for inflammatory fever.

## Highlights

• Intravenous lipopolysaccharide fails to induce fever in EP3-deficient mice.
• EP1, EP2, and neural EP4 deletion do not affect lipopolysaccharide-induced fever.
• EP3-deficient mice display marked hypothermia after intravenous lipopolysaccharide.
• EP3 is the only EP receptor uniquely required for fever.

## 1 Introduction

Fever induced by systemic inflammation is mediated by prostaglandin E2 (PGE2) synthesized by brain endothelial cells (Blomqvist, 2024; Shionoya et al., 2022), which acts on thermoregulatory circuits in the preoptic hypothalamus (Blomqvist and Engblom, 2018; Machado et al., 2020). Among the four known prostaglandin E (EP) receptors (Sugimoto and Narumiya, 2007), EP3 has been most strongly implicated in fever generation. Mice lacking EP3 display markedly attenuated febrile responses to lipopolysaccharide (LPS), interleukin-1β, and centrally administered PGE2 (Lazarus et al., 2007; Ushikubi et al., 1998), and EP3 receptors are abundantly expressed in thermoregulatory regions of the preoptic area (Ek et al., 2000; Nakamura et al., 2000). Furthermore, EP3-expressing neurons in the median preoptic region are critical for the febrile response (Lazarus et al., 2007).

Nevertheless, other EP receptor subtypes have also been implicated in the febrile response. In particular, mice deficient in the EP1 receptor were reported to exhibit markedly attenuated fever following systemic inflammation (Oka et al., 2003a), while pharmacological and local microinjection studies have implicated both EP1 and EP4 receptors in PGE2-evoked hyperthermia (Oka et al., 1997, 1998, 2003b; Osaka, 2022). Hence, although EP3 signaling is widely considered central for fever generation, the relative contribution of the different EP receptor subtypes has not been fully clarified.

Interpretation of previous studies is complicated by methodological differences, including variation in ambient temperature, genetic background, route of immune challenge, and injection-related disturbance of the animals, which may introduce stress-induced hyperthermia (Romanovsky et al., 1998). Such factors can substantially influence thermoregulatory responses and thereby complicate direct comparisons between studies. Furthermore, the different EP receptor-deficient mouse lines have not previously been examined side-by-side under identical experimental conditions.

In the present study, we therefore systematically compared febrile responses to intravenous LPS in mice lacking EP1, EP2, EP3, or EP4 receptors under carefully controlled conditions, including thermoneutral housing to minimize cold-defense thermogenesis, telemetry-based temperature recordings, and remote intravenous injections through indwelling jugular catheters, thereby avoiding handling of the animals during the injection procedure. Our results identify EP3 as the only EP receptor with a non-redundant role in LPS-induced fever.

## 2 Materials and methods

### 2.1 Animals

**Fig. 1:**
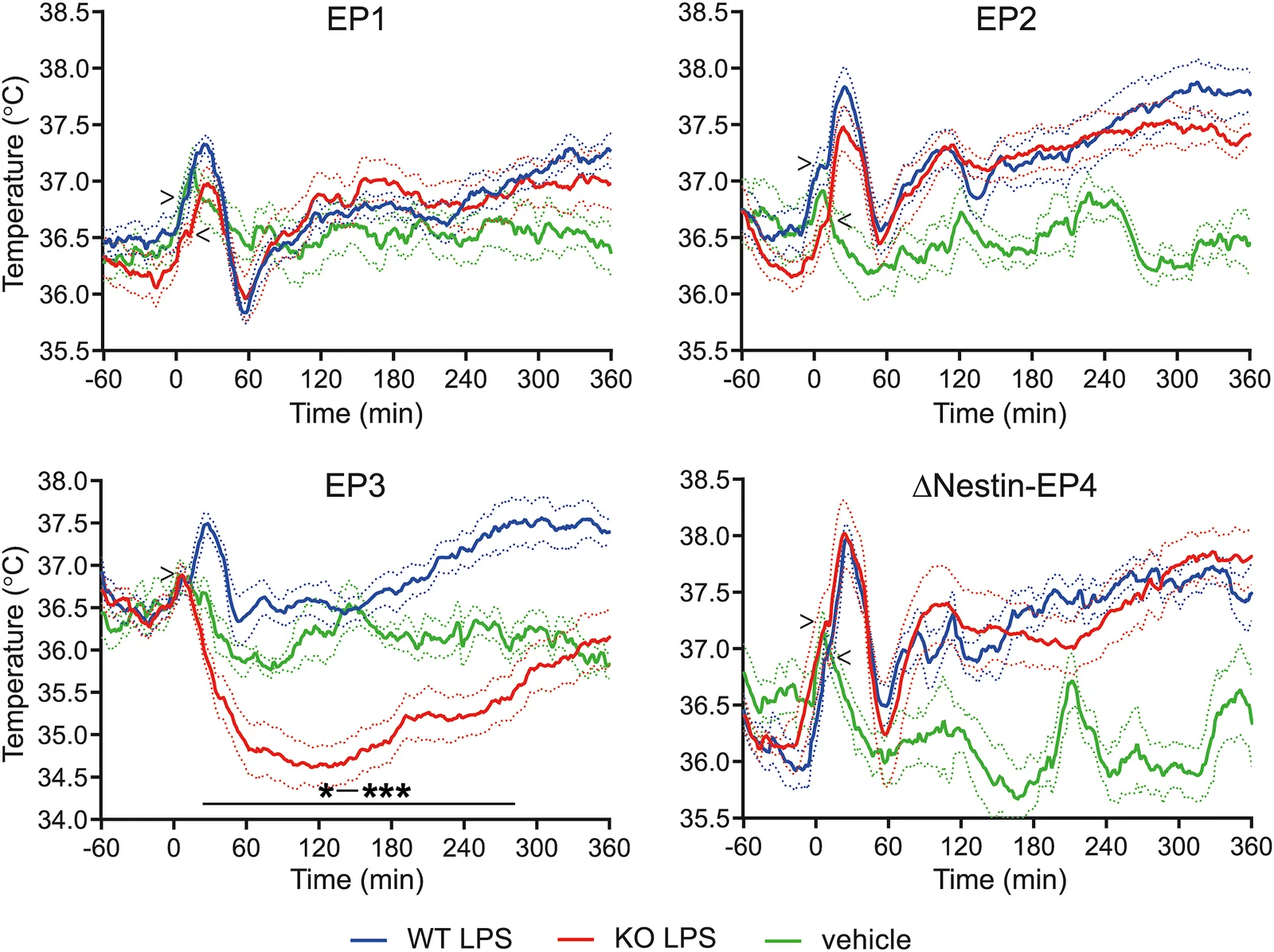
Intraperitoneal temperature recordings in WT and EP receptor-deficient mice following intravenous injection of lipopolysaccharide (30 μg/kg) or saline at time 0. Solid and dotted lines indicate means and SEM, respectively. Open arrowheads indicate the small notch in the rising temperature curve observed immediately after injection, probably reflecting a transient response to the intravenous injection. EP1: n = 8 (4F, 4M) for WT mice injected with LPS, n = 9 (4F, 5M) for KO mice injected with LPS, and n = 6 (4F, 2M) for saline-injected mice. EP2: n = 9 (5F, 4M) for WT mice injected with LPS, n = 9 (3F, 6M) for KO mice injected with LPS, and n = 6 (3F, 3M) for saline-injected mice. EP3: n = 8 (6F, 2M) for WT mice injected with LPS, n = 8 (5F, 3M) for KO mice injected with LPS, and n = 6 (4F, 2M) for saline-injected mice. ΔNestin-EP4: n = 5 (2F, 3M) for WT mice injected with LPS, n = 5 (2F, 3M) for KO mice injected with LPS, and n = 4 (1F, 3M) for saline-injected mice.

A total of 93 male and female mice aged 2–6 months were included. Group sizes ranged from 4 to 9 mice; exact group sizes and sex distributions are given in Fig. 1 legend. The study was not designed or powered to assess sex-dependent effects. Animals were housed under standard conditions at 21°C, except during postoperative recovery and experimentation, when they were kept at 29°C to provide near-thermoneutral conditions. They were maintained on a 12 h/12 h light/dark cycle (lights on at 7 a.m.), with food and water available ad libitum. All animal experiments were approved by the Linköping Animal Ethics Committee (approval nos. 35-11 and 20018-2023) and conducted in accordance with international guidelines.

Heterozygous mice carrying targeted deletions of EP1 (Stock et al., 2001), EP2 (Tilley et al., 1999), or EP3 (Fleming et al., 1998) (kindly provided by Dr. Beverly H. Koller) were maintained as heterozygotes on a C57BL/6 background and crossed to generate EP1-, EP2-, and EP3-deficient mice together with corresponding WT littermates. Because global EP4 deletion causes postnatal lethality due to persistent ductus arteriosus (Nguyen et al., 1997), neural EP4-deficient mice were generated by crossing homozygous mice carrying a conditional EP4 allele (EP4^fl/fl^) (Schneider et al., 2004) (kindly provided by Dr. Matthew D. Breyer), also on a C57BL/6 background, with EP4^fl/fl^ mice expressing Cre recombinase under the control of the Nestin promoter, which targets neurons and glia derived from neural progenitors (The Jackson Laboratory, Bar Harbor, ME, USA; strain 003771; Tronche et al., 1999). Littermate mice lacking the Nestin-Cre allele were used as controls (hereafter referred to as WT). Genotypes were determined by PCR of genomic DNA obtained from ear samples or tail samples when necessary.

### 2.2 Real-time PCR

To verify the efficiency and tissue specificity of EP4 deletion, mice with Nestin-Cre-mediated deletion of EP4 and their WT (EP4^fl/fl^) littermates (n = 5) were killed by carbon dioxide asphyxiation and perfused transcardially with saline to remove blood. The brain, kidneys, and spleen were dissected out, and the hypothalamus was isolated as described previously (Reyes et al., 2003). Tissues were immersed in RNAlater stabilization reagent (Qiagen, Hilden, Germany) and stored at −70°C until further processing. RNA was extracted using the RNeasy Universal Plus kit (Qiagen), and reverse transcription was performed with the High Capacity cDNA Reverse Transcription kit (Thermo Fisher, Waltham, MA, USA). Quantitative PCR was carried out using Gene Expression Master Mix (Thermo Fisher) in a 7900HT Fast Real-Time PCR system (Thermo Fisher). The following TaqMan assays were used: Mm00436053_m1 (*Ptger4*) and Mm99999915_g1 (*Gapdh*).

### 2.3 Surgery, immune challenge, and body temperature recording

Mice used for the temperature experiments were anesthetized with ketamine (70 mg/kg) and dexmedetomidine (0.4 mg/kg; both from Orion Pharma, Danderyd, Sweden), implanted intraperitoneally with a transponder for recording core body temperature (E-Mitter; Starr Life Sciences, Oakmont, PA, USA), and fitted with an indwelling jugular catheter. The catheter was exteriorized at the back of the neck and connected to a swivel system at the top of the cage, permitting intravenous injections without handling the mice (Engström et al., 2012). Following surgery, the mice were housed at an ambient temperature of 29°C, providing near-thermoneutral conditions (Rudaya et al., 2005). Four days later, at around 9 A.M., the mice were injected intravenously with lipopolysaccharide (LPS; Sigma-Aldrich, Burlington, MA, USA; O111:B4, 30 μg/kg) or saline through the indwelling catheter, and body temperature was recorded for approximately 8 h. LPS was dissolved in 5 μl saline and injected together with approximately 60–75 μl saline used to flush the dead space of the catheter system and ensure delivery of the LPS into the bloodstream. The LPS dose has been shown to produce a pronounced febrile response with largely distinct fever phases (Eskilsson et al., 2023; Shionoya et al., 2022; see also Rudaya et al., 2005).

### 2.4 Statistics

In all experiments, littermates were used. Groups were designed to be age- and sex-matched and, within these constraints, animals were randomly assigned to the respective groups. Statistical analyses were performed using Prism version 10 (GraphPad, Boston, MA, USA). qPCR data were analyzed using Student's t-test. Temperature recordings were analyzed by repeated-measures two-way ANOVA with group and time as factors, followed by post hoc testing with correction for multiple comparisons controlling the false discovery rate according to the linear step-up procedure. Differences were considered statistically significant at *P* < 0.05.

## 3 Results

Real-time PCR analysis showed that *Ptger4* expression in the hypothalamus was markedly reduced, to approximately 5–6% of WT levels (*P* < 0.001). To assess the specificity of the Nestin-Cre-mediated deletion, we also examined *Ptger4* expression in the kidney and spleen, two organs known to express EP4 receptors abundantly (Breyer and Breyer, 2001; Nataraj et al., 2001). In the kidney, *Ptger4* expression was reduced by approximately 40%, although this reduction did not reach statistical significance (P = 0.058), whereas no reduction was detected in the spleen. The renal reduction is consistent with reported off-target recombination in Nestin-Cre mice (Dubois et al., 2006), whereas the absence of an effect in spleen indicates sparing of the immune compartment examined here.

The body temperature recordings following intravenous injection of LPS or saline are shown in Fig. 1. LPS administration to WT mice generally evoked a multiphasic febrile response, with a rapidly developing first phase peaking around 20 min after injection, followed by a transient fall in body temperature with a nadir at approximately 60 min, and then second and third febrile phases, the latter emerging around 150–200 min after injection, although the transition between these phases was not always clearly demarcated.

As evident from the response to saline, temperature changes associated with the injection procedure were minimal, consisting only of a small and rapidly disappearing rise in body temperature occurring at the time of injection. This response, which likely reflects a transient physiological reaction to the intravenous injection itself, was also discernible in the LPS-injected WT, EP1-, EP2-, and EP4-deficient mice, where it appeared as a small notch in the rising temperature curve during the first febrile phase.

As shown in Fig. 1, mice with global deletion of EP1 or EP2, as well as mice with Nestin-Cre-mediated deletion of EP4, displayed body temperature responses to LPS that were indistinguishable from those of their WT littermates. In striking contrast, mice with global deletion of EP3 completely failed to develop fever and instead displayed an immediate and pronounced hypothermic response following LPS injection.

## 4 Discussion

The present study demonstrates that EP3 is the only prostaglandin E receptor with a non-redundant role in LPS-induced fever under standardized physiological conditions. Whereas mice lacking EP1 or EP2, or with nervous system-directed deletion of EP4, displayed febrile responses indistinguishable from those of their WT littermates, mice lacking EP3 completely failed to develop fever and instead displayed a pronounced hypothermic response immediately following LPS administration. Together with previous studies identifying endothelial PGE2 synthesis as necessary for fever generation (Shionoya et al., 2022), the present findings support a model in which EP3-expressing neurons constitute the critical thermoregulatory pathway downstream of inflammatory PGE2 signaling.

Impaired fever in EP3-deficient mice was previously demonstrated by Ushikubi et al. (1998) and later confirmed in mice with neural deletion of EP3 (Oishi et al., 2015). However, subsequent studies using pharmacological approaches and receptor-deficient mice suggested additional involvement of EP1 and EP4 in the febrile response (Oka et al., 1997, 1998, 2003a, 2003b; Osaka, 2022). Because these studies differed substantially in experimental conditions, the relative contribution of the individual EP receptor subtypes has not been fully clarified. The present study addresses this issue by directly comparing all receptor-deficient lines under identical experimental conditions.

A limitation is that EP4, unlike EP1–EP3, was examined using nervous system-directed rather than global deletion, precluding conclusions about EP4 expressed in other tissues. The partial reduction of renal *Ptger4* expression is unlikely to explain the absence of a febrile phenotype, although a functional effect of this off-target recombination cannot be excluded. In addition, the study was not powered to assess sex-dependent effects.

Methodological differences may explain discrepancies with previous reports. All receptor-deficient lines were examined under identical conditions with regard to genetic background, ambient temperature, route of immune challenge, and recording methodology. Furthermore, injections were administered remotely through indwelling jugular catheters, minimizing disturbance associated with the injection procedure. Such disturbance can itself evoke transient hyperthermia and interfere with reliable analysis of the full multiphasic febrile response (Romanovsky et al., 1998). The very small temperature response following saline injection in the present study indicates that these effects were minimal.

Interpretation of previous studies is further complicated by the fact that much of the evidence implicating EP1 and EP4 receptors has relied on pharmacological approaches or local microinjections of receptor agonists. Such experiments are limited by the incomplete selectivity of available EP receptor agonists and antagonists in vivo, particularly at the doses commonly used experimentally. Moreover, the ability of a receptor agonist to evoke hyperthermia after local administration does not necessarily imply that the same receptor is physiologically required for fever induced by systemic inflammation.

EP3 deletion unmasked an immediate and sustained hypothermic response following LPS administration, similar to that reported in mice lacking neural EP3 receptors (Oishi et al., 2015). This suggests that systemic inflammation activates thermoregulatory mechanisms promoting heat loss that are normally overridden by EP3-dependent febrile signaling, such that loss of EP3 shifts the response from fever toward hypothermia. Hypothermic responses to severe inflammatory challenge are well recognized in rodents and may represent an evolutionarily conserved energy-saving response (Liu et al., 2012).

In conclusion, the present findings identify EP3 as the only prostaglandin E receptor with a non-redundant role in LPS-induced fever. By directly comparing all four EP receptor subtypes under identical experimental conditions, the present study clarifies the role of prostaglandin receptor subtypes in fever generation and further strengthens the concept that EP3-expressing thermoregulatory neurons constitute the final common pathway for inflammatory fever.

## CRediT authorship contribution statement

**Unn Kugelberg:** Writing – review & editing, Methodology, Investigation. **Anna Erkstam:** Writing – review & editing, Investigation. **Kiseko Shionoya:** Writing – review & editing, Investigation. **Anders Blomqvist:** Writing – review & editing, Writing – original draft, Supervision, Methodology, Funding acquisition, Formal analysis, Conceptualization.

## Ethics declaration

This study was conducted in accordance with the ARRIVE (Animal Research: Reporting of In Vivo Experiments) guidelines. This study was approved by the Linköping Animal Ethics Committee. (Approval No. 35-11 and 20018-2023)

## Funding

This work was supported by the Swedish Brain Foundation (grant FO2025-0110).

## Declaration of competing interest

The authors declare that they have no known competing financial interests or personal relationships that could have appeared to influence the work reported in this paper.

## Data Availability

Data will be made available on request.
